# Combined RAF1 protein expression and p53 mutational status provides a strong predictor of cellular radiosensitivity

**DOI:** 10.1054/bjoc.2000.1409

**Published:** 2000-10

**Authors:** H M Warenius, M Jones, T Gorman, R McLeish, L Seabra, R Barraclough, P Rudland

**Affiliations:** Human Tumour Biology Group, University Clinical Departments, Oncology Research Unit, Department of Medicine, The University of Liverpool, The Duncan Building, Daulby Street, Liverpool, L69 3GA; Cancer and Polio Research Fund Laboratories, School of Biological Sciences, The University of Liverpool, Life Sciences Building, Crown Street, Liverpool, L69 7ZB, UK

**Keywords:** RAF1, p53, radiosensitivity, exit from G2/M

## Abstract

The tumour suppressor gene, p53, and genes coding for positive signal transduction factors can influence transit through cell-cycle checkpoints and modulate radiosensitivity. Here we examine the effects of RAF1 protein on the rate of exit from a G2/M block induced by γ-irradiation in relation to intrinsic cellular radiosensitivity in human cell lines expressing wild-type p53 (wtp53) protein as compared to mutant p53 (mutp53) protein. Cell lines which expressed mutp53 protein were all relatively radioresistant and exhibited no relationship between RAF1 protein and cellular radiosensitivity. Cell lines expressing wtp53 protein, however, showed a strong relationship between RAF1 protein levels and the radiosensitivity parameter SF2. In addition, when post-irradiation perturbation of G2/M transit was compared using the parameter T50 (time after the peak of G2/M delay at which 50% of the cells had exited from a block induced by 2 Gy of irradiation), RAF1 was related to T50 in wtp53, but not mutp53, cell lines. Cell lines which expressed wtp53 protein and high levels of RAF1 had shorter T50s and were also more radiosensitive. These results suggest a cooperative role for wtp53 and RAF1 protein in determining cellular radiosensitivity in human cells, which involves control of the G2/M checkpoint. © 2000 Cancer Research Campaign


*British Journal of Cancer* (2000) **83**(8), 1084–1095
© 2000 Cancer Research Campaign
doi: 10.1054/ bjoc.2000.1409, available online at http://www.idealibrary.com on IDEAL®

### Combined RAF1 protein expression and p53 mutational status provides a strong predictor of cellular radiosensitivity

**HM Warenius[1], M Jones[1], T Gorman[1], R McLeish[1], L Seabra[1], R Barraclough[2] and P Rudland[2]**

[1]Human Tumour Biology Group, Oncology Research Unit, Department of Medicine, The University of Liverpool, University Clinical Departments, The Duncan Building, Daulby Street, Liverpool L69 3GA; [2]Cancer and Polio Research Fund Laboratories, School of Biological Sciences, The University of Liverpool, Life Sciences Building, Crown Street, Liverpool L69 7ZB, UK

**Summary** The tumour suppressor gene, p53, and genes coding for positive signal transduction factors can influence transit through cell-cycle checkpoints and modulate radiosensitivity. Here we examine the effects of RAF1 protein on the rate of exit from a G2/M block induced by $\gamma$-irradiation in relation to intrinsic cellular radiosensitivity in human cell lines expressing wild-type p53 (wtp53) protein as compared to mutant p53 (mutp53) protein. Cell lines which expressed mutp53 protein were all relatively radioresistant and exhibited no relationship between RAF1 protein and cellular radiosensitivity. Cell lines expressing wtp53 protein, however, showed a strong relationship between RAF1 protein levels and the radiosensitivity parameter SF2. In addition, when post-irradiation perturbation of G2/M transit was compared using the parameter T50 (time after the peak of G2/M delay at which 50% of the cells had exited from a block induced by 2 Gy of irradiation), RAF1 was related to T50 in wtp53, but not mutp53, cell lines. Cell lines which expressed wtp53 protein and high levels of RAF1 had shorter T50s and were also more radiosensitive. These results suggest a cooperative role for wtp53 and RAF1 protein in determining cellular radiosensitivity in human cells, which involves control of the G2/M checkpoint. © 2000 Cancer Research Campaign

**Keywords**: RAF1; p53; radiosensitivity; exit from G2/M

A better understanding of the mechanisms underlying radio-responsiveness could help predict which patients are most likely to benefit from radiotherapy, and also holds the possibility of selectively modulating these mechanisms to improve the radiocurability of human cancer. Human in vitro cell lines of different histological origins which exhibit a range of intrinsic radiosensitivity, as measured by clonogenic cell survival assays, have been shown to provide appropriate models of the response of clinical tumours to radiotherapy (Fertil and Malaise, 1981; Deacon et al, 1984). We are therefore attempting to identify those genes whose protein expression is related to intrinsic radiosensitivity in a wide range of human in vitro cell lines. Such gene expression should prove to be relevant with regard to clinical radioresponsiveness.

The molecular basis of intrinsic radiosensitivity has been investigated for many years. A considerable body of research has focused on the degree of DNA damage and its subsequent repair as reflected in the incidence of double-strand breaks (dsbs) in the DNA (Kelland et al, 1988; Schwartz et al, 1991), the residual damage remaining in the DNA after cellular rejoining of dsbs (Nunez et al, 1995; Whitaker et al, 1995) and the fidelity of DNA repair (Powell and McMillan, 1994). In addition to DNA damage, however, it has become increasingly apparent that certain oncogenes and tumour suppressor genes may not only be implicated in carcinogenesis, but can also influence the sensitivity of malignant cells to ionizing radiation.

*Received 16 September 1999*
*Revised 13 June 2000*
*Accepted 28 June 2000*

*Correspondence to:* HM Warenius

Transfection of normal cell lines with dominant oncogenes such as *myc* and *ras* (McKenna et al, 1990) has resulted in increased radioresistance, even in the absence of detectable changes in the rate of dsb induction (Iliakis et al, 1990). Several other dominant oncogenes including c-*fms*, v-*sis*, v-*erb*-B, v-*abl*, v-*src*, v-*cot* (FitzGerald et al, 1990; Shimm et al, 1992; Suzuki et al, 1992) and in particular c-*raf*-1 (Kasid et al, 1987; 1989; Pirollo et al, 1989), have also been reported to modulate cellular radiosensitivity in mammalian cells. The potential relevance of these findings to clinical radiotherapy has been emphasized by our own observation that, in contrast to the c-*raf*-1 activated oncogene (Kasid et al, 1989), high levels of the normal protein product of the *RAF1* proto-oncogene are positively related to intrinsic radiosensitivity in human in vitro cell lines (Warenius et al, 1994). An additional body of evidence indicates a positive relationship between mutation in the p53 tumour suppressor gene and increased cellular radioresistance in both rodent and human tumour cells (Lee and Bernstein, 1993; Fan et al, 1994; Radford, 1994; Xia et al, 1995; Zhen et al, 1995) and in normal cells transfected with mutant p53 (mutp53) genes (Bristow et al, 1994; Pardo et al, 1994; Kawashima et al, 1995).

A number of reports suggest that oncogenes and tumour suppressor genes may modulate intrinsic radiosensitivity by their influence on the progress of irradiated cells through radiation-induced blocks at cell-cycle checkpoints. G1/S delay, mediated by p53 following exposure to ionizing radiation, has been implicated as an important measure of cell-cycle perturbation which correlates with relative radiation sensitivity (Kastan et al, 1991; McIlwrath et al, 1994; Siles, 1996). Also, the expression of dominant oncogenes such as *myc* and *ras* (McKenna et al, 1991) or SV40 T antigen (Su and Little, 1993) has been shown to induce both radioresistance and a concomitant increase in post-radiation delay at the G2/M checkpoint. We have previously demonstrated that radiosensitive human cancer cells, with high as opposed to low expression of the normal RAF1 protein, exhibit more rapid exit from a G2/M block induced by 2 Gy of radiation than radioresistant cells with low expression of RAF1 (Warenius et al, 1996).

Most studies up to the present have concentrated on the individual roles of oncogenes and tumour suppressor genes in modulating intrinsic radiosensitivity. When investigating the relationship between expression of a chosen gene and intrinsic radiosensitivity, consideration has not necessarily been given as to whether other candidate genes than the one selected for study might also have an affect on the outcome of experiments. This consideration could be particularly relevant in human clinical cancers, the majority of which exhibit multiple disorders of expression of oncogenes and suppressor genes. Moreover, little is known about whether, or how, human oncogenes and suppressor genes may interact to influence the radiosensitivity of cancer cells in the clinical situation. However, transfection experiments using cells from other mammals, such as rat embryo fibroblasts (REF), have demonstrated greater increases in radioresistance in cells expressing dominant plus cooperating oncogenes than expressing the single dominant oncogenes alone (McKenna et al, 1990; Pirollo et al, 1993; Su and Little, 1993). Similarly, radioresistance induced in REF cells by transfection with multiple-integrated mutant p53-pro193 alleles was much greater when the mutant p53 gene was co-transfected with H-*ras* (Bristow et al, 1994). In the human in vitro cell lines which we are using as a model system, there is considerable disorder of gene expression, and there is no clear relationship between the patterns of protein expression of different oncogenes or proto-oncogenes. We have therefore not been able to investigate the putative influence of co-expression of oncogenes on intrinsic radiosensitivity at the protein level. In order to progress to a better understanding of the influence of multiple genes on radiosensitivity and the possible interaction of proto-oncogenes and suppressor genes, we have, therefore, investigated the effect of the mutational status of the p53 gene on our previously-described relationship between RAF1 protein, radiosensitivity and exit from a radiation induced G2/M cell-cycle checkpoint block.

### MATERIALS AND METHODS

### Cell lines and clonogenic cell survival assays

The growth characteristics, irradiation and clonogenic assay procedures and radiation cell survival of the 12 human in vitro cell lines in which the p53 mutational status has now been determined, have already been reported (Warenius et al, 1994). The cell lines are listed, with their histological classification in Table 1. All are well established, many having been growing in vitro for several years. Cell lines were either donations or purchased by our laboratories. On receipt all were grown for five passages to provide sufficient cells for batch storage in liquid nitrogen. During this period, contamination was excluded by at least one passage in antibiotic-free medium, and mycoplasma testing was carried out on all lines. For clonogenic assays, cells were taken from a designated primary liquid nitrogen batch and grown for 3–6 passages until there were sufficient well-growing cells. Further batches from these cells were frozen in liquid nitrogen. Cells were routinely maintained in DMEM medium except RT112 and H322, which were grown in RPMI1640 and MGHU-1 which were grown in Ham's F12 medium. All lines were supplemented with 10% heat-inactivated fetal calf serum (HIFCS).

As previously described (Warenius et al, 1994; 1996), the radiosensitivity of the 12 human lines described here was obtained from radiation cell survival curves consisting for the majority of cell lines of 6–12 dose points over the range 0–6 Gy and then at 1 Gy intervals to 8–10 Gy, as appropriate. For each cell line repeated assays were performed until sufficient data were obtained to give statistically acceptable values for $\alpha$ (the initial slope of the radiation cell survival curve) and $\beta$ when fitted by the linear quadratic equation (below). Following irradiation from a 4 MeV linear accelerator, at 2 Gy $min^{-1}$ or a $^{137}Cs$ Gammacell unit at 4 Gy $min^{-1}$, exponentially growing cells were seeded at densities from $10^2$ to $5 \times 10^4$ in 5 ml of Ham's F12 medium plus 10% FCS in 60 mm petri dishes and incubated at 37°C in 5% $CO_2$ for 10–14 days. The cells were then fixed in 70% ethanol, stained with 10% Giemsa and colonies of greater than 100 cells counted. Cell-survival curves were obtained by fitting the experimental data points with the linear quadratic equation:

$$S = e^{-(\alpha D + \beta D^2)}$$

**Table 1** Mutations detected in the p53 gene and consequent amino-acid changes in 7/12 human cell lines

| Cell line[a] | Source | cDNA sequence[b] | Amino-acid change | p53 protein[c] |
|---|---|---|---|---|
| 1407 | Embryonic intest. epith. | Normal | none | Wild-type |
| HEP 2 | Squamous carcin. larynx | Normal | none | Wild-type |
| MGHU 1 | Transit. carcinoma bladder | Normal | none | Wild-type |
| HRT18 | Adenocarcinoma rectum | Normal | none | Wild-type |
| 2780 | Ovarian carcinoma | Normal | none | Wild-type |
| OAW 42 | Ovarian carcinoma | CGA–CGG codon 213 | none | Wild-type |
| HT 29/5 | Adenocarcinoma colon | CGT–CAT codon 273 | Arg–His | Mutant |
| COLO 320 | Adenocarcinoma colon | CGG–TGG codon 245 | Arg–Tryp | Mutant |
| H 322 | Small cell carcinoma lung | CGG–TGG codon 245 | Arg–Tryp | Mutant |
| H 417 | Small cell carcinoma lung | GAG–TAG codon 298 | Glu–Stop | Truncated |
| RPMI 7951 | Melanoma | TCA–TTA codon 166 | Ser–Stop | Truncated |
| RT 112 | Transit. carcinoma bladder | CCG–CAG codon 248 | Arg–Gly | Mutant |

[a] Origin and growth conditions described in Materials and Methods; [b] Sequencing was carried out by the dideoxynucleotide enzymatic method using the fmol DNA Sequencing System, as described in Materials and Methods; [c] As predicted by cDNA sequence

*© 2000 Cancer Research Campaign*

where S is the surviving fraction at dose D and α and β are constants. The data were fitted to the equation using a Graphpad Prism program (GraphPad Software, San Diego, CA, USA). SF2 values (the surviving fraction at 2 Gy) were obtained by interpolation of the fitted cell-survival curves.

### Identification of mutations in the p53 gene by PCR and DNA sequencing

Material for PCR and DNA sequencing of p53 and Western blotting for RAF1 protein, was obtained from the same liquid nitrogen batches used to provide cells for clonogenic cell-survival data. Cells were grown for up to three passages prior to being subjected to the following procedures.

### *Nucleic acid isolation*

RNA and genomic DNA were prepared from the cell lines described here by the guanidinium isothiocyanate CsCl gradient method (Chirgwin et al, 1979; Barraclough et al, 1987). Briefly, the cells were collected in ice-cold phosphate-buffered saline (PBS) and homogenized in guanidinium isothiocyanate buffer (4M guanidinium isothiocyanate, 50 mM Tris pH 7.5, 25 mM EDTA pH 8.0, 0.5% (w/v) sodium lauryl sarcosine and 8% (v/v) 2-mercaptoethanol) added just prior to use. The homogenate was cleared by centrifugation at 8000 rpm for 10 min at 4°C (SS34 rotor, Sorvall RC-5B centrifuge) and the RNA pelleted by centrifugation of the homogenate through a cushion of 5.7 M CsCl 0.1 M EDTA at 32 000 rpm for 20 h at 20°C (TST 41.14 rotor, Kontron Centrikon T20 60 ultracentrifuge). The pellet of RNA was redissolved in 0.1% (w/v) SDS and precipitated with ethanol overnight at –20°C before quantitation.

### *Polymerase chain reaction, cDNA synthesis and nucleotide sequencing*

PCR (for exons 2–8 and for exons 9–11) was performed on DNA and RNA extracted from the 12 human carcinoma cell lines. Each exon was then examined by DNA sequencing. PCR primers were designed flanking each exon and synthesized on an Applied Biosystems 381A DNA synthesizer. Each exon was amplified separately with the exceptions of exons 2 and 3 which were amplified as a unit, and exons 9, 10 and 11 which were amplified together by reverse transcription polymerase chain reaction (RT-PCR). The following primers were used:

Exon 2/3 sense 5′-CCC ACT TTT CCT CTT GCA GC-3′
Exon 2/3 antisense 5′-AGC CCA ACC CTT GTC CTT AC-3′
Exon 4 sense 5′-CTG CTC TTT TCA CCC ATC TA-3′
Exon 4 antisense 5′-GCA TTG AAG TCT CAT GGA AG-3′
Exon 5 sense 5′-TGT TCA CTT GTG CCC TGA CT-3′
Exon 5 antisense 5′-CAG CCC TGT CGT CTC TCC AG-3′
Exon 6 sense 5′-GCC TCT GAT TCC TCA CTG AT-3′
Exon 6 antisense 5′-TTA ACC CCT CCT CCC AGA GA-3′
Exon 7 sense 5′-ACT GGC CTC ATC TTG GGC CT-3′
Exon 7 antisense 5′-TGT GCA GGG TGG CAA GTG GC-3′
Exon 8 sense 5′-T ATC CTG AGT AGT GG-3′
Exon 8 antisense 5′-T GCT TGC TTA CCT CG-3′
Exon 9/10/11 sense 5′-AGA AAG GGG AGC CTC ACC AC-3′
Exon 9/10/11 antisense 5′-CTG ACG CAC ACC TAT TGC AA-3′

Genomic DNA was digested with EcoR1, precipitated with ethanol and resuspended in 50 µl of water (Sigma) before being subjected to PCR amplification. The DNA (1 µg) was amplified in 50 µl PCR reactions containing 20 pmoles of each primer. A 'hot-start' PCR protocol was used with the dNTPs and Taq enzyme initially separated from the rest of the reaction components on a wax cushion. The reactions were placed in a pre-heated PCR block at 95°C for 2 min before undergoing 30 cycles of denaturation (30 s at 95°C), annealing (30 s at 60°C for exons 2–3, 4 and 6; 65°C for exons 5 and 8; 67°C for exon 7; and 68°C for exon 9–11) and extension (1 min at 72°C). The PCR products were checked on a 0.8% (w/v) agarose gel before being purified using a Wizard minicolumn (Promega), and used directly in sequencing reactions.

### *cDNA synthesis and RT-PCR*

Complementary DNA was synthesized from approximately 5 µg of total RNA using oligo(dT) as a primer. Total RNA (5 µg), human placental ribonuclease inhibitor (HPRI) 20 U and 1 µg oligo (dT) were heated at 70°C for 10 min, chilled on ice, added to 1 × first-strand buffer (50 mM Tris-HCl, pH 8.3, 75 mM KCl and 3 mM MgCl), 0.01 M DTT, dNTPs (0.5 µM for each deoxyribonucleoside triphosphate), 400 U of superscript reverse transcriptase (Gibco) and incubated at 37°C for 1 h. PCR for exons 9–11 was carried out using 5 µl of the above incubation in a 50 µl of PCR reaction, as described in the previous section.

### *Nucleotide sequencing*

Sequencing primers (10 pmoles) were radioactively labelled at their 5′ ends with $\gamma^{32}$P-ATP (45 µCi) at 37°C for 30 min in a reaction containing T4 polynucleotide kinase (PNK) (9.7 U, Pharmacia) and 1 × T4 PNK buffer (10 µM Tris-acetate, 10 µM magnesium acetate and 50 µM potassium acetate). The primers used were identical to those employed in the PCR reactions except for exon 5, for which a separate sense sequencing primer was designed as follows: 5′-TAC TCC CCT GCC CTC-3′. Sequencing was carried out by the dideoxynucleotide enzymatic method (Sanger et al, 1997), using the fmol DNA Sequencing System (Promega). Any putative sequence mutations identified were confirmed by additional sequencing of the exon in the antisense direction as well as by carrying out a repeat PCR and sequencing of the cell line.

### *Western blotting for RAF1*

Two independent Western blottings with lysates for each cell line, loaded in pairs on each gel, were carried out. $10^7$ cells were grown in 162 cm$^2$ tissue culture flasks (Costar Ltd, High Wycombe, UK) until they were pre-confluent and still growing exponentially. Cells were then removed by trypsinization, resuspended in complete medium plus 10% FCS and washed three times by serial centrifugation and resuspension in PBS without serum. 1–3 × $10^8$ viable cells were then pelletted by centrifugation and resuspended at 3 × $10^7$ cells per ml of lysis buffer (× 1 stock solution: 10% (w/v) SDS 10 ml, 0.5 M Tris, pH 6.8, glycerol 10 ml, double-distilled water 62 ml; to 10 ml of stock solution were added 100 µl of 10 µM leupeptin plus 10 µl 100 µM PMSF). Protein estimations were performed and the final concentration of the lysates adjusted to 300 µg total cellular protein per 100 µl of lysis buffer. To measure RAF1 protein, 150 µg of total cellular protein in 50 µl of lysate buffer was added per lane to a 7.5% Laemmli separating gel and electrophoresis carried out at 16°C using 60 V over 16 h and a constant current of 500 mA. Proteins were transferred to nitrocellulose at 22°C over 16 h using a semi-dry blotting apparatus (Biorad, Richmond, CA, USA), the membrane incubated with the

 © 2000 Cancer Research Campaign

URP26S3 mouse monoclonal antibody to human RAF1 (kindly donated by Dr Ulf Rapp) and then with rabbit anti-mouse conjugated antibodies (Dako, UK) at 1/1000 and developed in alkaline phosphatase buffer containing Nitroblue Tetrazolium 75 mg $ml^{-1}$ in 70% (v/v) DMF and 5-bromo-4-chloro-3-indoyl phosphate (Sigma, Poole, Dorset, UK) (50 mg $ml^{-1}$ in dimethylformamide). Quantitation of the full length 72–74 kD RAF1 protein product of the *RAF1* proto-oncogene was carried out by measurement of optical density on a Shimadzu scanning densitometer with tungsten light and expressed as OD units per 150 μg of total cellular protein. Titration curves obtained by loading different amounts of total cellular protein have previously shown that linear relationships for optical density (OD) could be obtained over the range found for RAF1 protein across the cell lines (Warenius et al, 1994; Browning, 1996). In order to compare different levels of RAF1 protein between the cell lines, the mean OD value for all the lines was calculated and the relative OD for RAF1 protein in each individual cell line was normalized to the mean OD and multiplied by an arbitrary value of 5.0.

### *Measurement of exit from radiation-induced G2/M arrest (T50)*

All experiments were carried out on asynchronous, exponentially growing cultures. Progress through G2/M following the relatively low radiation dose of 2 Gy was followed because we were interested in the relative effects of radiation compared to RAF1 levels and cell-cycle checkpoint perturbation at clinically relevant doses (Warenius et al, 1994; 1996). Cells were plated at a density of 1.5–2 × $10^5$ cells in 25 $cm^2$ flasks (Costar) in 8 ml of normal medium on the day before irradiation. Aliquots of cells were plated in pairs of flasks for each time-point. Once the cells had attached, the flasks were completely filled with medium containing 10% (v/v) FCS and 20 mM HEPES and incubated at 37°C overnight. One of each pair of flasks was either sham-irradiated (control in Figure 1) or exposed to 2 Gy of γ-radiation from a $^{137}Cs$ gammacell unit at 4 Gy $min^{-1}$. Care was taken to minimize any fall in temperature during this stage.

### *Flow cytometry*

Following irradiation the flasks were returned to the incubator and subsequently harvested by trypsinization at 2 h intervals, washed once in PBS, fixed in 70% (v/v) ethanol and stored at 4°C. For DNA content analysis, fixed cells were pelleted and washed once by centrifugation followed by resuspension in PBS containing 20 μg $ml^{-1}$ propidium iodide (PI) and 100 μg $ml^{-1}$ RNase A. Samples were incubated for at least 30 min before being filtered through a 41 μm nylon filter (Spectrum, Texas, USA) and then analysed on a Becton Dickinson FACS 420 flow cytometer using 488 nm illumination and a 620 nm long-pass filter. Collected list mode data was analysed using the ModfFit cell-cycle analysis software (Verity, Maine, USA). This enabled the relative distribution of cells in the G1, S and G2/M phases of the cell cycle to be determined. Three experimental runs each recording 2 h time-points for 24 h for both irradiated cells and sham-irradiated controls were carried out for each cell line. Data were plotted and linear regression analyses carried out using a Fig P programme and non-parametric analyses were performed with an Arcus package.

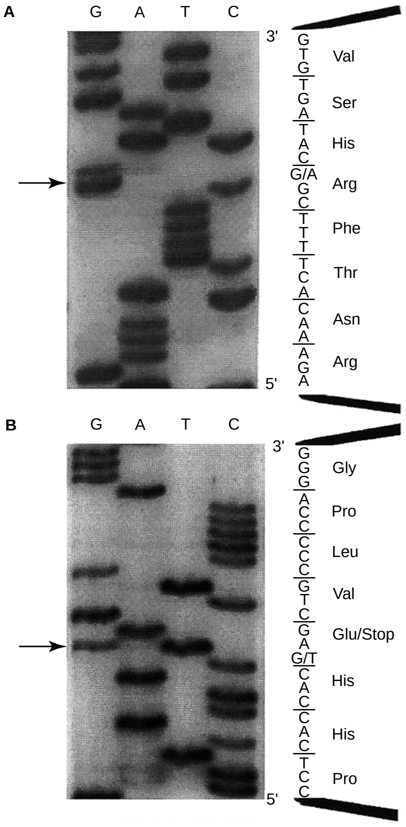

**Figure 1** Regions of p53 cDNA dideoxy sequencing gels showing point mutations in (**A**) OAW42 human ovarian cancer and (**B**) H417 human small cell lung cancer cell lines

### RESULTS

Mutations were found in mRNA expressed from the p53 gene in seven of the 12 cell lines. The mutations identified in the cell lines described here were in exons 5–8, which are known to contain the majority of p53 mutations (Hollstein et al, 1991). All these mutations have been previously described, apart from the nonsense mutation identified in codon 166 of the RPMI7951 line. This along with the G–T transversion in codon 298 of H417 did not lie within the most highly conserved region of the p53 gene. In the OAW42 ovarian carcinoma cell line the single-base missense mutation from CGA–CGG was silent, so that the mutant triplet still coded for the same amino acid (Arg) as is present in wtp53 protein (Figure 1A). A normal p53 protein was thus expressed in six of the 12 cell lines. The mRNA of the other six cell lines coded for abnormal p53 protein (Table 1). RPMI7951 and H417 possessed stop mutations resulting in 165 and 297 amino acid-truncated proteins, respectively. COLO320 and H322 independently exhibited a missense G–C to A–T mutation at the same site, resulting in an amino-acid substitution from Arg to Tryp. RT112 and HT29/5 also had mutations coding for changes in Arg (to Gly and His, respectively). COLO320, H322 and RT112 were homozygous for p53 mutations. The other three mutant lines showed evidence of retention of heterozygosity (as shown for H417 in Figure 1B). HT29/5 and RPMI7951 both expressed small amounts of wtp53 mRNA although H417 expressed relatively high levels. 50% of

*© 2000 Cancer Research Campaign*

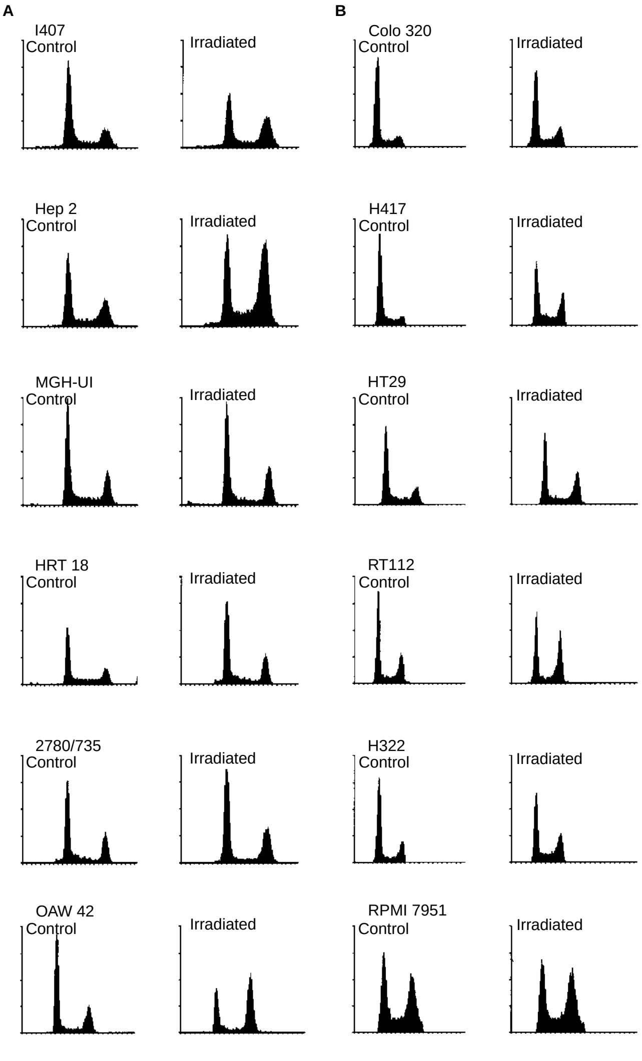

**Figure 2** Flow cytometric histograms of propidium iodide (PI)-labelled human in vitro cancer cell lines 12 h after exposure to 2 Gy of ionizing radiation. Ordinate fluorescent intensity. Abcissa cell number

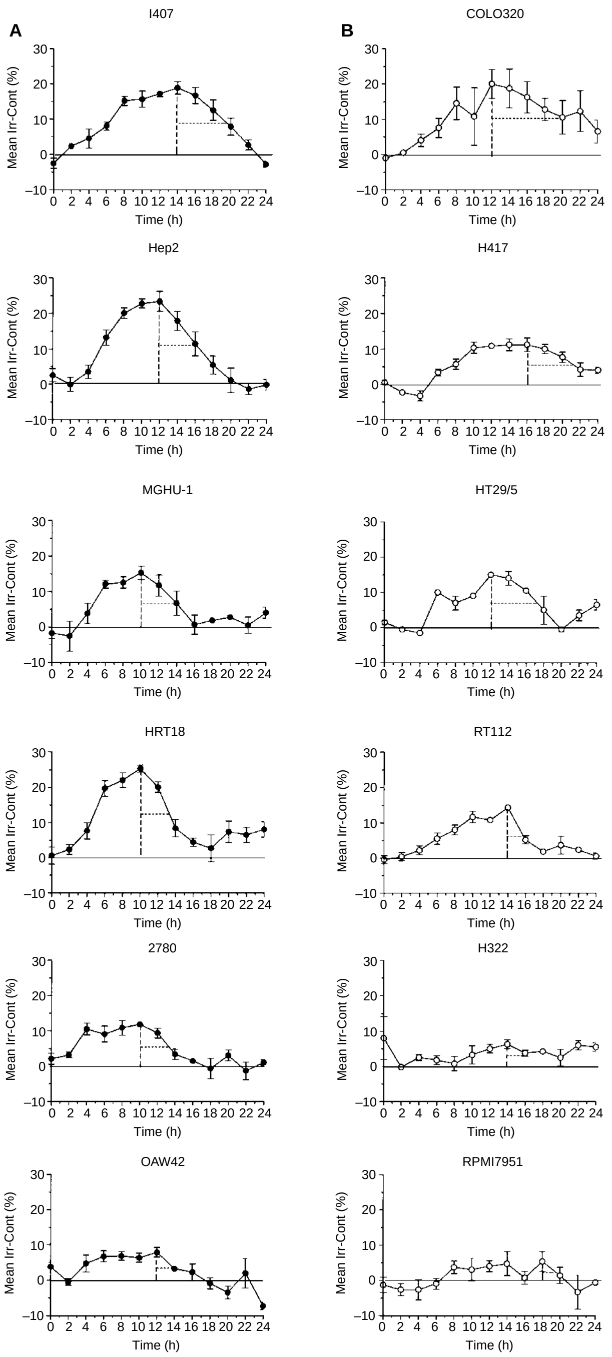

**Figure 3** Percentages of cells in the G2/M phase of the cell cycle obtained by analysis of serial PI histograms over a period of 24 h following exposure to 2 Gy of ionizing radiation. For each 2-h time-point, the value of the sham-irradiated cells was subtracted from that of its irradiated counterpart and the mean ± 1 SEM of a minimum of three consecutive experiments was calculated (**A**) wtp53, (**B**) mutp53. Vertical dotted line is peak of G2 delay. Horizontal dotted line indicates time for 50% of cells to exit from peak G2/M delay (T50)

our lines contained the residue 72 polymorphism which has been recently described as playing an important role in the development of human papilloma-virus-associated cancer (Storey et al, 1998). These were evenly distributed between wild-type and mutant p53 cell lines.

The percentage of cells in the G2 phase of the cell cycle was measured by flow cytometry at serial 2 h time-points following exposure to 2 Gy of γ-irradiation, as previously described (Warenius et al, 1996). No differences in the quality of the DNA histograms of PI-stained cells were distinguishable between wtp53 and mutp53 cells (Figure 2). Post-irradiation G2 delay in mutp53 cells occurred around the same time-points of 10–14 h and was generally less marked than in wtp53 cells (Figure 3). Otherwise radiation-induced G2 delay appeared to be little affected by p53 mutational status per se. In both the wtp53 and mutp53 groups of cell lines, a range from slight to marked radiation-induced G2 delay was observed. The degree of G2 delay induced by 2 Gy of ionizing radiation was quantified as described previously (Warenius et al, 1996) by measuring the rate of exit from the G2 block (expressed as T50 (Table 2)). Although the T50 values for mutp53 cell lines exhibited a higher mean (3.88) and range (1.29–7.77) than the T50 values for wtp53 (mean 3.42, range 1.85–4.99), both sets of values fully overlapped and no significant differences in T50 were apparent between the two groups of cell lines.

The relationship between RAF1 protein levels, intrinsic radiosensitivity (SF2), and the rate of exit from a radiation-induced G2 block was examined for all 12 cell lines and then independently in

*© 2000 Cancer Research Campaign*

**Table 2** RAF1 protein, radiosensitivity and post-irradiation G2 exit in (**A**) wtp53 and (**B**) mutp53 human cell lines

| Cell line[a] | RAF1 protein[b] | Intrinsic radiosensitivity[c] SF2 | Post-irradiation G2 exit[d] T50 (h) |
|---|---|---|---|
| **Wtp53** | | | |
| I 407 | 3.33 ± 1.31 | 0.54 | 4.99 |
| HEP 2 | 3.82 ± 0.65 | 0.65 | 4.07 |
| MGHU 1 | 5.27 ± 0.67 | 0.61 | 3.70 |
| HRT18 | 5.94 ± 0.38 | 0.41 | 3.33 |
| 2780 | 9.25 ± 1.16 | 0.30 | 2.59 |
| OAW 42 | 10.39 ± 1.16 | 0.22 | 1.85 |
| **Mutp53** | | | |
| HT 29/5 | 0.65 ± 0.18 | 0.59 | 5.18 |
| COLO 320 | 5.44 ± 0.90 | 0.34 | 7.77 |
| H 322 | 3.58 ± 0.17 | 0.53 | 2.03 |
| H 417 | 8.46 ± 1.03 | 0.48 | 5.55 |
| RPMI 7951 | 4.20 ± 0.43 | 0.56 | 1.29 |
| RT 112 | 5.04 ± 0.91 | 0.60 | 1.48 |

[a] Origin and growth conditions described in Materials and Methods; [b] Arbitrary OD units ± 1 SEM calculated as described in Materials and Methods; [c] SF2 values obtained by interpolation of cell survival curves fitted by the linear quadratic equation to experimental cell survival data as described in Materials and Methods; [d] T50 values obtained from experimental data depicted in Figure 3 as described in Materials and Methods the wtp53 and mutp53 cells. Typical Western blots for RAF1 protein and p53 protein in the twelve cell lines are shown in Figure 4 at 0 and 8 h after irradiation with 2 Gy. No obvious changes were apparent in the Western blots in any of the cell lines as a result of irradiation for p53 or RAF1. The ranges of RAF1 protein levels in wtp53 cells and mutp53 cells overlapped (3.33–10.39 and 3.58–8.46, respectively, Table 2), though the highest RAF1 values were seen in the wtp53 cell lines. While RAF1 protein levels were strongly correlated with SF2 for all 12 cell lines ($r$ = –0.77, $P$ = 0.004, Figure 5), in keeping with previous observations (Warenius et al, 1994; 1996), no overall correlation was found between the rate of G2 exit (T50) and RAF1 level ($r$ = –0.19, $P$ = 0.55) or between T50 and SF2 ($r$ = –0.04, $P$ = 0.91).

Comparison of the wtp53 cell lines to the mutp53 cell lines, however, (Figure 6) indicated that the mutual correlation between radiosensitivity, a short T50 and high RAF1 protein expression was only found in wtp53 cells. A highly significant negative relationship between RAF1 protein levels and T50 in wtp53 cells (r = –0.97, $P$ = 0.002) (Figure 6C) which was not present in the six cell lines bearing p53 mutations ($r$ = 0.19, $P$ = 0.72) (Figure 6F) was observed. A strong negative correlation between RAF1 protein and SF2 in wtp53 cells ($r$ = –0.92, $P$ = 0.01, Figure 6A) was also found. There was a similar, though weak trend in mutp53 cells ($r$ = –0.46, $P$ = 0.36, Figure 6D). In the six wtp53 cell lines there was a significant positive correlation between SF2 and T50 ($r$ = 0.84, $P$ = 0.04, Figure 6B), whereas the mutp53 group of cell lines showed a non-significant correlation in the opposite direction ($r$ = –0.77, $P$ = 0.08, Figure 6E). Although the p53 mutant cell lines were in general more radioresistant, it was not possible to separate radiosensitive from radioresistant cell lines on the basis of p53 mutational status alone. This was because, as seen in Table 2, the SF2 values for wtp53 cells (range 0.22–0.54) overlapped with those of mutp53 cells (range 0.34–0.60).

In order to be confident of our results it is important that the means by which we have measured G2 exit takes into account factors that could impact upon the ability of the T50 values to show a correlation with SF2, and therefore also with RAF1 in the mutant cell lines. For example, the rate of delivery of cells to G2 and the possible non-movement of cells through the cell cycle due to cell death could potentially affect G2 accumulation. For this reason, we chose to examine only the rate of exit from G2/M as this was least likely to be influenced by factors occurring earlier in the cell cycle. We have noted, however, in several of the cell lines presented here, that when visualized under time-lapse microscopy there is negligible cell death prior to the first mitosis (unpublished data). In addition, when sequential changes in the G1 and S-phase populations of the 12 cell lines were examined, it could be seen that there was no evidence of early post-radiation impairment of G1/S transit in wtp53 or mp53 cells (Figure 7) which might have

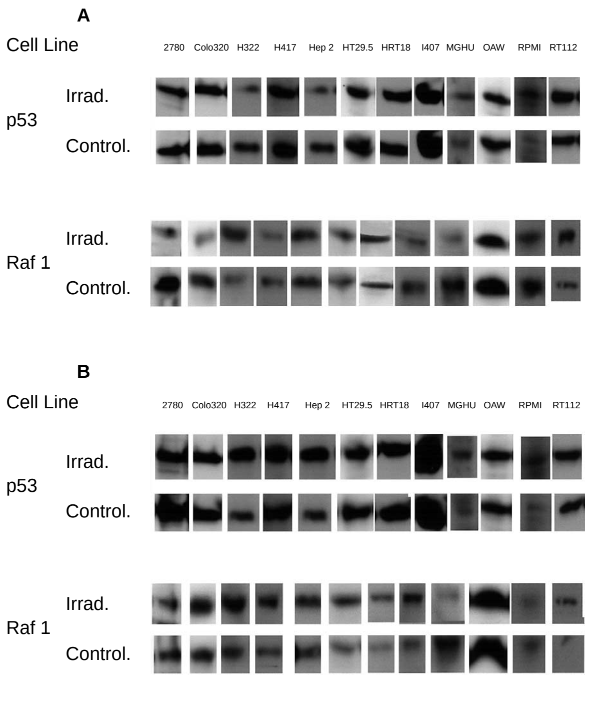

**Figure 4** Typical Western blots of 12 human cell lines (**A**) immediately and (**B**) 8 h following 2 Gy of gamma irradiation.

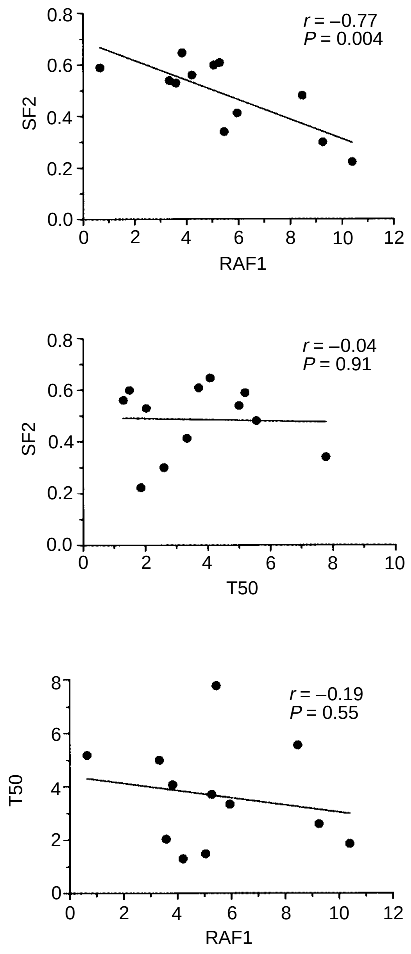

**Figure 5** Relationship between RAF1 protein levels, radiosensitivity (SF2) and rate of exit from a G2/M block following 2 Gy of γ-radiation (T50) for all 12 human cell lines irrespective of p53 mutational status (data from Table 2)

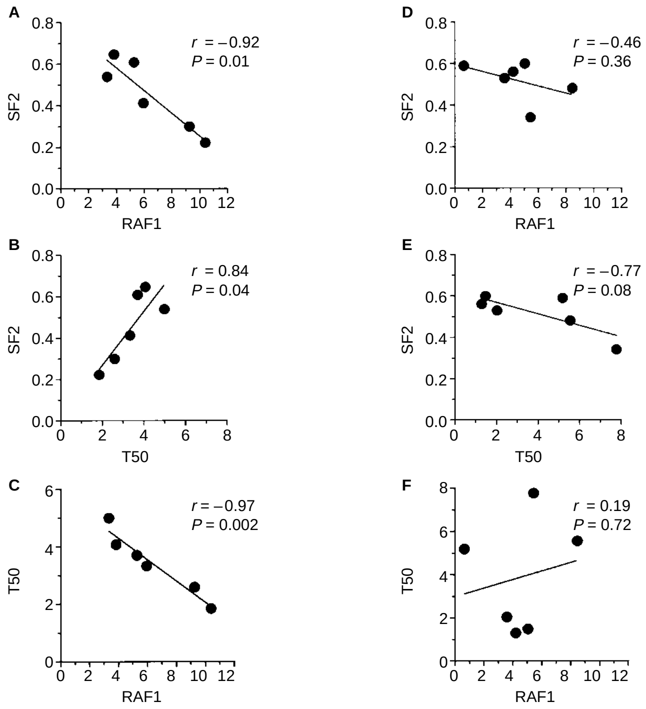

**Figure 6** Comparison of relationships between RAF1, radiosensitivity and rate of exit from a post-irradiation G2/M block in p53 wild-type (**A, B, C**) and p53 mutant (**D, E, F**) cell lines (data from Table 2)

potentially given rise to a different rate of delivery of cells to G2 in the mutant as compared to the wild-type p53 populations. In both wtp53 and mutp53 cells the first post-radiation cell-cycle change observed was a small accumulation of cells in S-phase, indicating a block of S-phase exit into G2 (Figure 8). The S-phase block had cleared by around 4 h prior to the peak accumulation of cells in G2/M in both groups and was thus unlikely to influence the G2/M exit T50 value. In order to be certain that the results presented here were not influenced by different cell-cycle transit times in the wtp53 and mutp53 populations, we compared the values for potential doubling times (Tpots) for each group. Wild-type p53 cells had a mean Tpot of 20.47 (range 9.69–29.94) and mutant p53 cells had a mean Tpot of 20.01 (range 15.33–32.01) indicating closely similar cell-cycle transit times in the two groups of cells. In addition, Tpots were compared to T50 values in each group of cells. It can be seen that there were no significant correlations between Tpots and T50s in either the mutant or wild-type p53 cells (Figure 9).

Serial changes in p53 and RAF1 protein levels were measured over the 24-h periods during which cell-cycle changes were recorded by flow cytometry (Figure 10). In all cell lines p53 and RAF1 changes tended to move together, but no patterns of change consistent with changes in the cell cycle were seen for either protein and there were no obvious differences between mutant and wild-type p53 cells.

### DISCUSSION

Here we show that the relationship we have previously identified between RAF1 expression and relative radiosensitivity can be clearly demonstrated in human cell lines which express mRNA coding for wtp53 protein but is absent in mutp53 cell lines. The mutations detected in 7/12 of the cell lines are generally typical of p53 mutations previously reported in human cancers. Many reports investigating the mutational status of the p53 gene have concentrated on sequencing exons 5–9 as this region is known to contain the majority of mutations (Hollstein et al, 1991). However, published data covering a wide range of cancers has estimated that up to 6% of p53 mutations can occur outside exons 5–9 (de Fromentel and Soussi, 1992). In order to be certain that we were accurately assigning our cell lines as p53 mutants or wild-type, we therefore chose to sequence the whole of the coding sequence of the gene (exons 2–11). The Arg 273 and Arg 248 mutations we identified occur at two of the known mutational hotspots in human cancer and are residues that interact directly with DNA. The codon 245 Arg mutation has been previously reported in colon and lung carcinoma cell lines (Rodrigues et al, 1990; Takahashi et al, 1991). The codon 213 silent mutation in the OAW42 cell line is the site of a polymorphism found in approximately 3.5% of the population (Carbone et al, 1991; Mazars et al, 1992). A silent point mutation at this site has also been described in breast carcinomas and osteosarcomas (McIntyre et al, 1994).

© 2000 Cancer Research Campaign

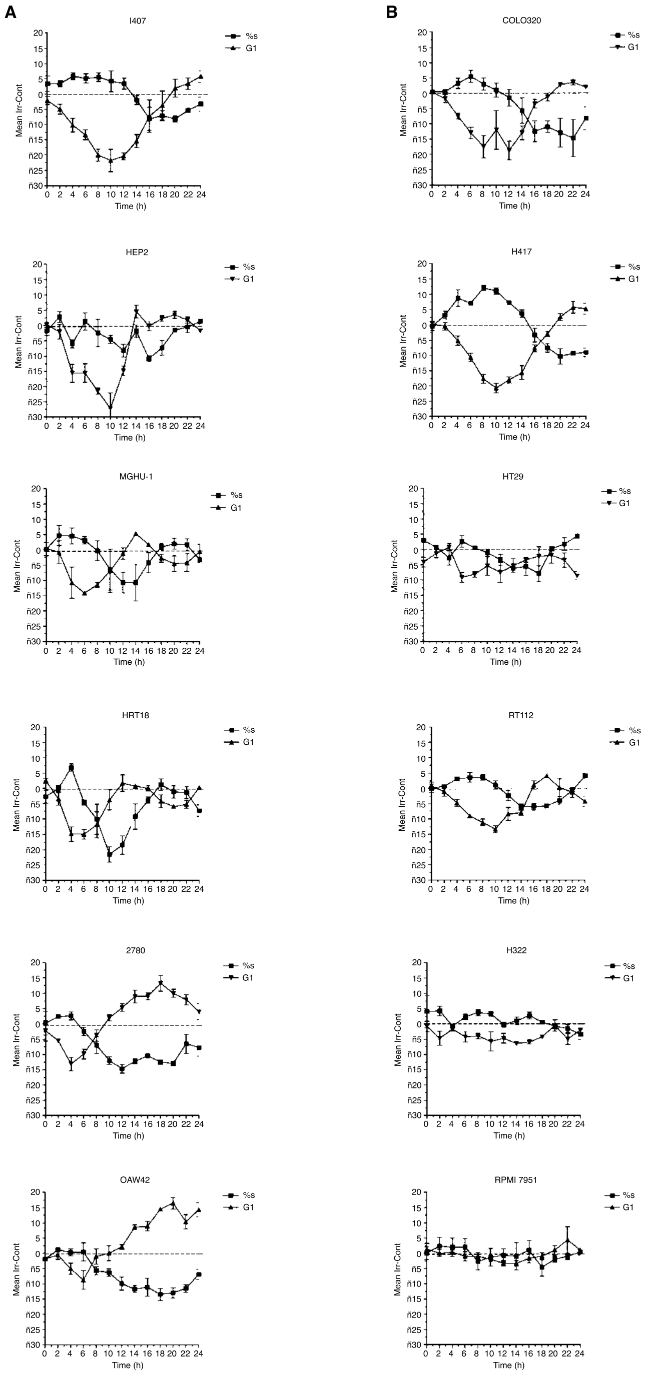

**Figure 7** Percentage of cells in the $G_1$ and S phases of the cell cycle over 24 h obtained by analysis of serial PI histograms after exposure to 2 Gy gamma irradiation (at time 0). For each 2-h time-point the value of the sham-irradiated cells was subtracted from that of its irradiated counterpart and the mean ± 1 SEM of a minimum of three consecutive experiments was calculated (**A**) wtp53 (**B**) mutp53

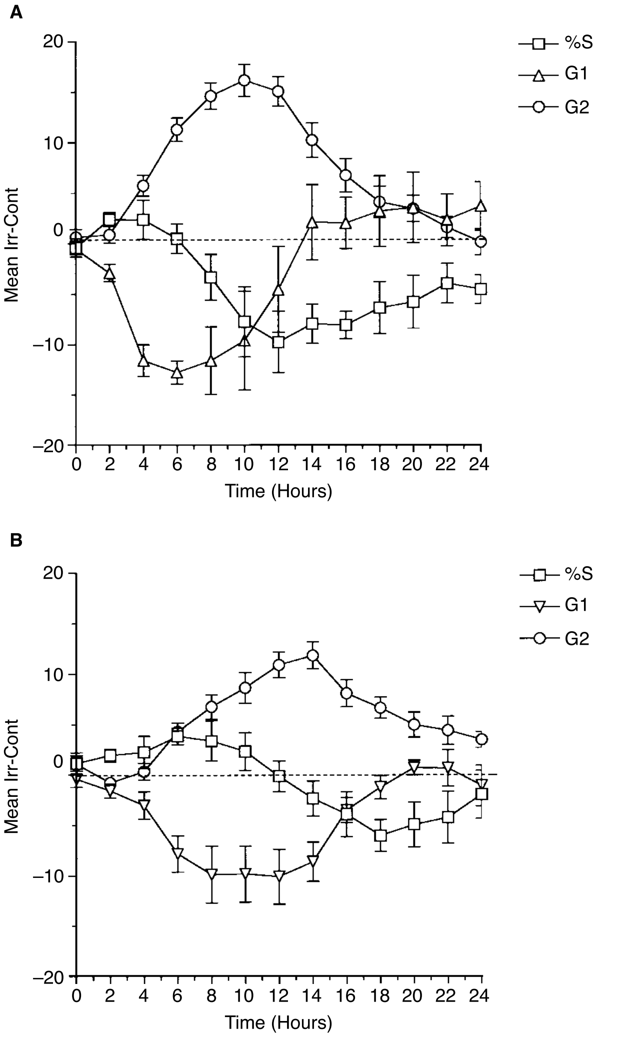

**Figure 8** Composite data from Figures 3 and 7 to illustrate overall time courses for each cell-cycle phase change in $G_1$, S and $G_2$/M after 2 Gy of γ-irradiation. Each 2-h time-point is the mean of means ± 1 SEM for each of the (**A**) six wild-type and (**B**) six mutant cell lines

The identification of a very significant correlation between RAF1 and intrinsic radiosensitivity in wtp53 cells, which was absent in mutp53 cells (Figure 5A and 5B), indicates the potential importance of examining the expression of more than a single gene when attempting to relate gene expression and radioresponsiveness in human cancer cells with heterogenous genotypes. Considerable heterogeneity of gene expression is widely acknowledged to be present in human cancers in the clinical situation. The difficulty in extrapolating from simple experimental systems to complex human diseases has been emphasized by Tada et al (1998), who have reported that p53 mutations in cerebral glioblastomas are surprisingly strongly associated with radioresponsiveness rather than resistance to radiation therapy. The disparity of conclusions in clinicopathological studies which have attempted to correlate the status of the p53 gene alone to clinical outcome following radiotherapy (reviewed by Bristow et al, 1996) may reflect the lack of knowledge of the contemporaneous expression of other radiosensitivity-determining genes in the genetically heterogenous material obtainable from patients undergoing treatment for cancer. In these clinicopathological studies, radioresponsiveness was examined in relation to p53 mutational status determined by DNA sequencing or to the levels of p53 protein detected by immunocytochemistry. We have carried out Western blotting for p53 protein in the 12 lines described here using the CM1 antibody (unpublished data).

*© 2000 Cancer Research Campaign*

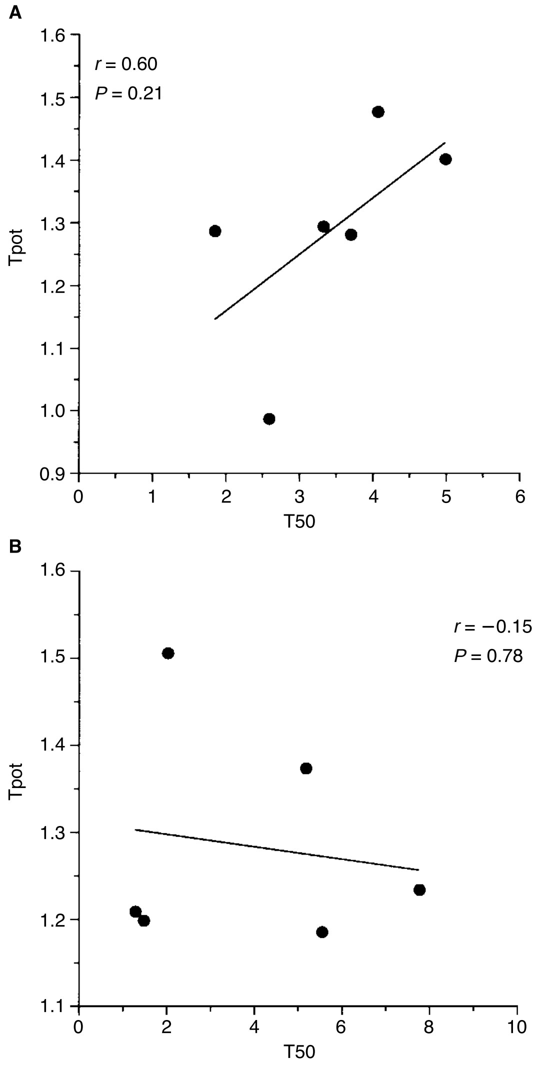

**Figure 9** Comparison of Tpot and T50 values for (**A**) wild-type and (**B**) mutant p53 cell lines fitted by linear regression

The p53 protein was detectable in all cells except the RPMI 7951 melanoma where the epitope recognized by CM1 was absent in the truncated protein. No relationship between the level of p53 protein and intrinsic radiosensitivity was detected. This finding is not unsurprising, as it would be expected that the functionality of the p53 protein, rather than the cellular concentration, would be of most importance in relation to the effect of p53 on radiosensitivity. In the cell lines examined here, those cells which contained p53 mutations and thus produced dysfunctional p53 proteins were, in general, relatively radioresistant with SF2 values of 0.34–0.6. Several other factors apart from structural abnormalities in the p53 gene may, however, influence its function. The cellular response to ionizing radiation may be dependent on transcriptional activation of the p53 gene which has been observed to be impaired in Fanconi anaemia (Rosselli et al, 1995) and ataxia telangiectasia (Kastan et al, 1992; Khanna and Lavin, 1993). In addition, p53 function has been shown to be regulated by post-translational factors including those modifying its stability (Midgley and Lane, 1997) or its phosphorylation status. Several proteins have been demonstrated to be capable of phosphorylating p53. These include MAP kinase (Milne et al, 1994), DNA-activated protein kinase (Lees Miller et al, 1992), casein kinase II (Milne et al, 1992), CDK1 (Bischoff et al, 1992) and CDK2, which transactivate as well as phosphorylate the p53 protein (Segawa et al, 1993). Recently it has been reported that phosphorylation of the ser15 residue within the N-terminal transactivating region of the p53 protein has been observed following exposure to ionizing radiation (Siliciano et al, 1998). It is possible that functional changes in wtp53 protein due to interactions with other molecules may provide a mechanism for the modulation of sensitivity to anti-cancer agents. A study of Blagoskonny et al (1995) indicates that induction of p53 and p21WAF1 in MCF7 human breast cancer cells by Taxol has been shown to be dependent on the expression of c-RAF1. To our knowledge there is at present no information regarding the modulation of radiosensitivity by the interaction of RAF1 and p53. However, activated RAF1 has been shown to phosphorylate and transactivate mouse p53 in the N-terminal transactivation domain, possibly on ser18, which is the equivalent residue to ser15 in the human p53 protein (Jamal and Ziff, 1995).

Our results are not consistent with a similar mechanism operating in the cell lines examined here. This is because, although time-course experiments following the fates of p53 and RAF1 by Western blotting showed that, in several cell lines, the two appeared to move together, there was no clear correlation between these post-radiation protein changes and radiosensitivity or G2/M exit. Moreover, p53 and RAF1 did not co-immunoprecipitate or show any evidence of phosphorylation on Western blotting. It is thus possible that the statistical relationship which we have shown between RAF1 expression and p53 status may be explained by other factors that do not involve a direct p53/RAF1 interaction, possibly involving p21WAF1/CIP. The results that we have observed here, however, indicate that further experiments are required to determine what mechanism may operate in wtp53 human in vitro cell lines which make RAF1 protein levels such a strong correlator with radiosensitivity and G2/M exit. We are at present co-transfecting certain of the wtp53 cell lines with a temperature-sensitive p53 mutant and both full-length and truncated activated RAF1 kinase genes under inducible-promoter control to investigate RAF1/wtp53 involvement in radiosensitivity and G2/M checkpoint control under isogenic conditions.

Not only do the levels of RAF1 protein relate strongly to intrinsic radiosensitivity in the wtp53 human cell lines examined here, but also both intrinsic radiosensitivity and RAF1 correlate with the rate of exit of wtp53 cells, but not mutp53 cells, from a G2/M block induced by 2 Gy of ionizing radiation (Figures 5B, 5C, 5E and 5F). These mutual inter-relationships suggest the possibility that RAF1 and wtp53 may be influencing intrinsic cellular radiosensitivity by interacting, in some as yet unknown way, at the G2/M cell-cycle checkpoint. Lovric and Moelling (1996) have reported five- to six-fold increased activation of RAF1 kinase activity in the mitotic phases of the cell cycle of a range of cell lines. This was accompanied by hyperphosphorylation of serine/threonine residues in the carboxy terminus of the RAF1 protein. They have suggested that activation of c-RAF1 at the G2/M boundary might be involved in p53 regulation, thereby providing a mechanism by which RAF1 influences cellular radiosensitivity. The association between high levels of RAF1 protein, relative radiosensitivity and rapid G2 exit in wild-type p53 cells seen in Figure 5 is consistent with this hypothesis and also with reports that transfection of wild-type p53 was able to induce

 © 2000 Cancer Research Campaign

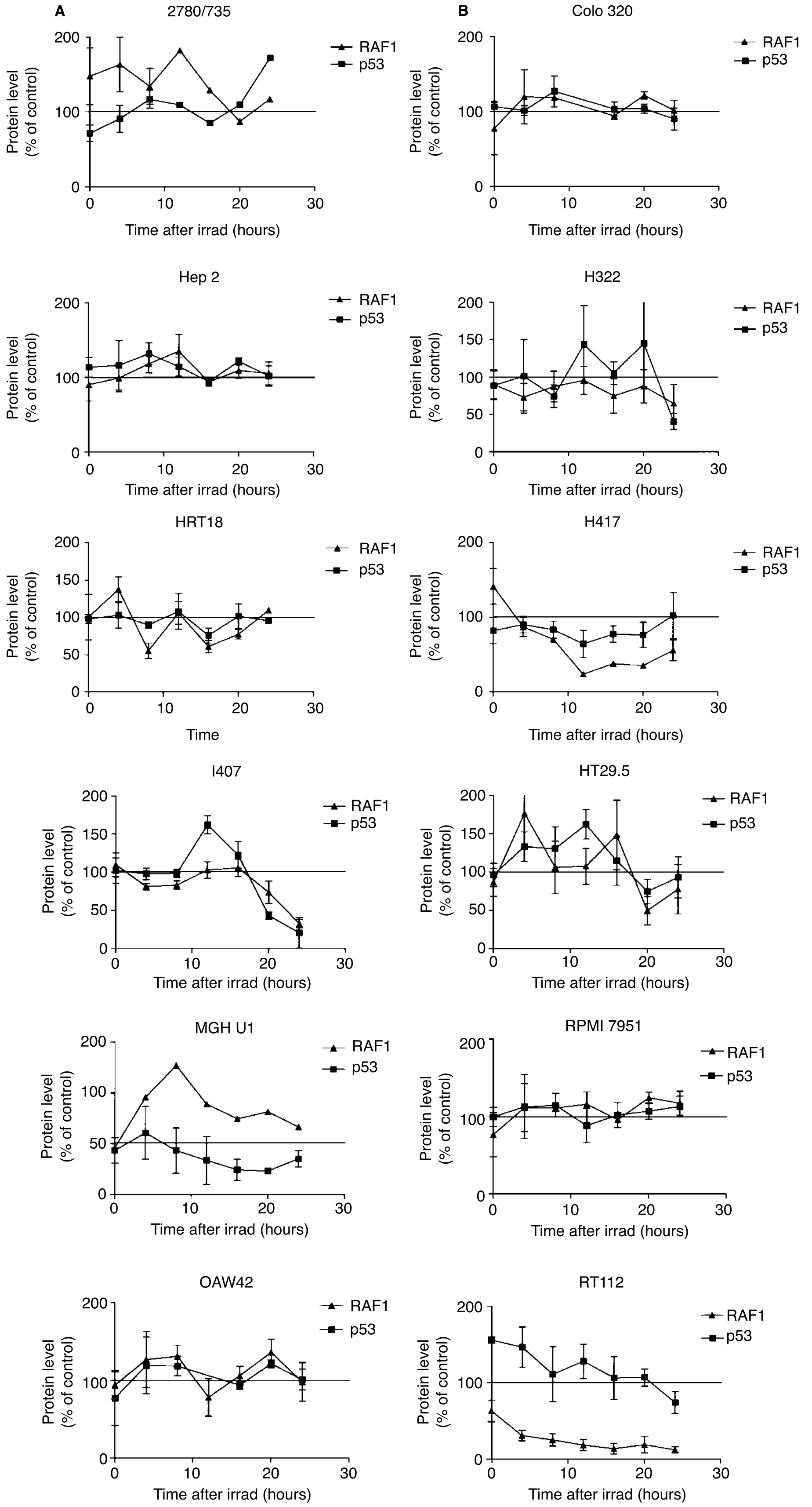

**Figure 10** Serial measurements of p53 and RAF1 protein levels by quantitative Western blotting over 24 h following 2 Gy of gamma irradiation. Each time-point is the mean of three independent experiments ± 1 SEM (**A**) wtp53, (**B**) mutp53

© 2000 Cancer Research Campaign radiosensitivity in association with rapid exit from a radiation-induced G2/M block in two independent experimental systems (Guillouf et al, 1995; Schwartz et al, 1997). However, several studies have described increased transit through the G2/M checkpoint in association with loss of wild-type p53 function (Aloni-Grinstein et al, 1995; Paules et al, 1995; Stewart et al, 1995). In order to explain these apparent discrepancies concerning the action of p53 at the G2/M checkpoint, Schwartz et al (1997) have suggested that in some cases p53 may arrest passage of cells through G2/M until DNA damage is repaired, while in other situations p53 may facilitate repair of DNA damage and hasten progress through G2/M. The net effect of these processes may depend on the type of DNA damage, the level of p53 expression and the type of cell under investigation (Schwartz et al, 1997). We have sought to minimize these variables by investigating the relationships of RAF1, p53 mutational status, the G2/M checkpoint and intrinsic radiosensitivity in a range of human in vitro cell lines which potentially allow the identification of general patterns of gene expression relevant to clinical therapeutic responsiveness against a heterogenous genetic background similar to that encountered in the patient. In the cell lines described here, the rate of exit from a G2/M block evoked by 2 Gy of γ-irradiation was not related to p53 mutational status per se, nor was p53 expression or mutational status a predictor of radiosensitivity. The high correlation we have obtained between SF2 and the level of expression of the protein product of the RAF1 proto-oncogene in wild-type p53 cells, however, could potentially provide a useful test for detecting the radioresponsiveness of certain tumours at dose levels commonly employed in the clinic.

### ACKNOWLEDGEMENTS

We are grateful to Mrs Carole Thomas for organising technical facilities and to Mrs Amanda Howarth for data analysis and preparation of diagrams and manuscript. This work was supported by The Cancer and Polio Research Fund, Hoylake, Merseyside and The North West Cancer Research Fund.

### REFERENCES

Aloni-Grinstein R, Schwartz D and Rotter V (1995) Accumulation of wild-type p53 protein upon γ-irradiation induces a $G_2$ arrest-dependent immunoglobulin κ light in chain gene expression. *EMBO J* **7**: 1392–1401

Barraclough R, Kimbell R and Rudland PS (1987) Differential control of mRNA levels for Thy-1 antigen and laminin in rat mammary epithelial and myoepithelial-like cells. *J Cell Physiol* **131**: 393–340

Bischoff JR, Casso D and Beach D (1992) Human p53 inhibits growth in Schizo saccharomyces pombe. *Mol Cell Biol* **12**: 1405–1411

Blagosklonny MV, Schulte TW, Nguyen P, Mimnaugh EG, Trepel J and Neckers L (1995) Taxol induction of $p21^{WAF1}$ and p53 requires c-*raf*-1. *Cancer Res* **55**: 4623–4626

Bristow RG, Jang A, Peacock J, Chung S, Benchimol S and Hill RP (1994) Mutant p53 increases radioresistance in rat embryo fibroblasts simultaneously transfected with HPV16-E7 and/or activated H-*ras*. *Oncogene* **9**: 1527–1536

Bristow RG, Benchimol S and Hill RP (1996) The p53 gene as a modifier of intrinsic radiosensitivity: implications for radiotherapy. *Radiother Oncol* **40**: 197–223

Browning PGW (1996) *Proto-oncogene expression and intrinsic cellular radiosensitivity*. PhD dissertation. The University of Liverpool: Liverpool, UK

Carbone D, Chiba I and Mitsudomi T (1991) Polymorphism at codon 213 within the p53 gene. *Oncogene* **6**: 1691–1692

Chirgwin JM, Przybyla AE, MacDonald RJ and Rutter WJ (1979) Isolation of biologically active ribonucleic acid from sources enriched in ribonuclease. *Biochemistry* **18**: 5294–5299

Deacon J, Peckham MJ and Steel GG (1984) The radioresponsiveness of human tumours and the initial slope of the cell survival curve. *Radiother Oncol* **2**: 317–323

de Fromentel C and Soussi T (1992) TP53 tumour suppressor gene – a model for investigating human mutagenesis. *Genes Chromosomes Cancer* **4**: 1–15

Fan S, el Deiry WS, Bae I, Freeman J, Jondle D, Bhatia K, Fornace AJ Jr, Magrath I, Kohn KW and O'Connor PM (1994) p53 gene mutations are associated with decreased sensitivity of human lymphoma cells to DNA damaging agents. *Cancer Res* **54**: 5824–5830

Fertil B and Malaise EP (1981) Inherent cellular radiosensitivity as a basic concept for human tumor radiotherapy. *Int J Radiat Oncol Biol Phys* **7**: 621–629

FitzGerald TJ, Henault S, Sakakeeny M, Santucci MA, Pierce JH, Anklesaria P, Kase K, Das I and Greenberger JS (1990) Expression of transfected recombinant oncogenes increase radiation resistance of clonal hematopoietic and fibroblast cell lines selectively at clinical low dose rate. *Radiat Res* **122**: 44–52

Guillouf C, Rosselli F, Krishnaraju K, Moustacchi E, Hoffman B and Liebermann DA (1995) p53 involvement in control of G2 exit of the cell cycle: role in DNA damage-induced apoptosis. *Oncogene* **10**: 2263–2270

Hollstein M, Sidransky D, Vogelstein B and Harris C (1991) p53 mutations in human cancers. *Science* **253**: 49–53

Iliakis G, Metzger L, Muschel RJ and McKenna WG (1990) Induction and reapir of double strand breaks in radiation-resistant cells obtained by transformation of primary rat embryo cells with the oncogenes H-ras and v-myc. *Cancer Res* **50**: 6575–6579

Jamal S and Ziff EB (1995) Raf phosphorylates p53 *in vitro* and potentiates p53-dependent transcriptional transactivation *in vivo*. *Oncogene* **10**: 2095–2101

Kasid U, Pfeifer A, Weichselbaum RR, Dritschilo A and Mark GE (1987) The *raf* oncogene is associated with a radiation-resistant human laryngeal cancer. *Science* **237**: 1039–1041

Kasid U, Pfeifer A, Brennan T, Beckett M, Weichselbaum RR, Dritschilo A and Mark GE (1989) Effect of antisense c-*raf*-1 on tumorigenicity and radiation sensitivity of a human squamous carcinoma. *Science* **243**: 1354–1356

Kastan MB, Onyekwere O, Sidransky D, Vogelstein B and Craig RW (1991) Participation of p53 protein in the cellular response to DNA damage. *Cancer Res* **51**: 6304–6311

Kastan MB, Zhan Q, el-Deiry WS, Carrier F, Jacks T, Walsh WV, Plunkett BS, Vogelstein B and Fornace AJ Jr (1992) A mammalian cell cycle checkpoint pathway utilising p53 and GADD45 is defective in ataxia-telangiectasia. *Cell* **71**: 587–597

Kawashima K, Mihara K, Usuki H, Shimizu N and Namba M (1995) Transfected mutant p53 gene increases X-ray-induced cell killing and mutation in human fibroblasts immortalized with 4-nitroquinoline 1-oxide but does not induce neoplastic transformation of the cells. *Int J Cancer* **61**: 76–79

Kelland LR, Edwards SM and Steel GG (1988) Induction and rejoining of DNA double-strand breaks in human cervix carcinoma cell lines of differing radiosensitivity. *Radiat Res* **116**: 526–538

Khanna KK and Lavin MF (1993) Ionizing radiation and UV induction of p53 protein by different pathways in ataxia-telangiectasia cells. *Oncogene* **8**: 3307–3312

Lee JM and Bernstein A (1993) p53 mutations increase resistance to ionizing radiation. *Proc Natl Acad Sci USA* **90**: 5742–5746

Lees Miller SP, Sakaguchi K, Ullrich SJ, Appella E and Anderson CW (1992) Human DNA-activated protein kinase phosphorylates serines 15 and 37 in the amino-terminal transaction domain of human p53. *Mol Cell Biol* **12**: 5041–5049

Lovric J and Moelling K (1996) Activation of Mil/Raf protein kinases in mitotic cells. *Oncogene* **12**: 1109–1116

Mazars GR, Jeanteur P, Lynch HT, Lenoir G and Theillet C (1992) Nucleotide sequence polymorphism in a hotspot mutation region of the p53 gene. *Oncogene* **7**: 781–782

McIlwrath AJ, Vasey PA, Ross GM and Brown R (1994) Cell cycle arrests and radiosensitivity of human tumor cell lines: dependence on wild-type p53 for radiosensitivity. *Cancer Res* **54**: 3718–3722

McIntyre JF, Smith-Sorensen B, Friend SH, Kassell J, Borrensen AL, Yan YX, Russo C, Sato J, Barbier N, Miser J, Malkin D and Gebhardt MC (1994) Germline mutations of p53 tumour suppressor gene in children with osteosarcoma. *J Clin Oncol* **12**: 925–930

McKenna WG, Weiss MC, Endlich B, Ling CC, Bakanauskas VJ, Kelsten ML and Muschel RJ (1990) Synergistic effect of the *v-myc* oncogene with H-*ras* on radioresistance. *Cancer Res* **50**: 97–102

McKenna WG, Iliakis MC, Weiss EJ, Bernhard EJ and Muschel RJ (1991) Increased $G_2$ delay in radiation-resistant cells obtained by transformation of primary rat embryo cells with the oncogenes H-ras and v-myc. *Radiat Res* **125**: 283–287

 *© 2000 Cancer Research Campaign*

Midgley CA and Lane DP (1997) p53 protein stability in tumour cells is not determined by mutation but is dependent on Mdm2 binding. *Oncogene* **15**: 1179–1189

Milne DM, Palmer RH and Meek DW (1992) Mutation of the casein kinase II phophorylation site absolishes the anti-proliferative activity of p53. *Nucleic Acids Res* **20**: 5565–5570

Milne DM, Campbell DG, Caudwell FB and Meek DW (1994) Phosphorylation of the tumour suppressor protein p53 by mitogen activated protein kinases. *J Biol Chem* **269**: 9253–9260

Nunez MI, Villalobos M, Olea N, Valenzuela MT, Pedraza V, McMillan TJ and Ruiz de Almodovar JM (1995) Radiation-induced DNA double-strand break rejoining in human tumour cells. *Br J Cancer* **71**: 311–316

Pardo FS, Su M, Borek C, Preffer F, Dombkowski D, Gerweck L and Schmidt EV (1994) Transfection of rat embryo cells with mutant p53 increases the intrinsic radiation resistance. *Radiat Res* **140**: 180–185

Paules RS, Levedakou EN, Wilson SJ, Innes CL, Rhodes N, Tlsty TD, Galloway DA, Donehower LA, Tainsky MA and Kaufmann WK (1995) Defective $G_2$ checkpoint function in cells from individuals with familial cancer syndromes. *Cancer Res* **55**: 1763–1773

Pirollo KF, Garner R, Yuan SY, Li L, Blattner WA and Chang EH (1989) *raf* involvement in the simultaneous genetic transfer of the radioresistant and transforming phenotypes. *Int J Radiat Biol* **55**: 783–796

Pirollo KF, Tang YA, Villegas Z, Chen Y and Chang EH (1993) Oncogene-transformed NIH 3T3 cells display radiation resistance levels indicative of a signal transduction pathway leading to the radiation-resistant phenotype. *Radiat Res* **135**: 234–243

Powell SN and McMillan TJ (1994) The repair fidelity of restriction enzyme-induced double strand breaks in plasmid DNA correlates with radioresistance in human tumor cell lines. *Int J Radiat Oncol Biol Phys* **29**: 1035–1040

Radford IR (1994) p53 status, DNA double-strand break repair proficiency and radiation response of mouse lymphoid and myeloid cell lines. *Int J Radiat Biol* **66**: 557–560

Rodrigues NR, Rowan A, Smith MEF, Kerr IB, Bodmer WF, Gannon JV and Lane DP (1990) p53 mutations in colorectal cancer. *Proc Natl Acad Sci USA* **87**: 7555–7559

Rosselli F, Ridet A, Soussi T, Duchaud E, Alapetite C and Moustacchi E (1995) p53-dependent pathway of radio-induced apoptosis is altered in Fanconi anemia. *Oncogene* **10**: 9–17

Sanger F, Nicklen S and Coulson AR (1997) DNA sequencing with chain terminating inhibitors. *Proc Natl Acad Sci USA* **74**: 5463–5467

Segawa K, Hokuto J, Minowa A, Okyama K and Takano T (1993) Cyclin E enhances p53 mediated transactivation. *FEBS Lett* **329**: 283–286

Schwartz JL, Mustafi R, Beckett MA, Gyzewski EA, Farhangi E, Grdina DJ, Rotmensch J and Weichselbaum RR (1991) Radiation-induced DNA double-strand break frequencies in human squamous cell carcinoma cell lines of different radiation sensitivities. *Int J Radiat Biol* **59**: 1314–1352

Schwartz D, Almog N, Peled A, Goldfinger N and Rotter V (1997) Role of wild type p53 in the $G_2$ phase: regulation of the γ-irradiation-induced delay and DNA repair. *Oncogene* **15**: 2597–2607

Shimm DS, Miller PR, Lin T, Moulinier PP and Hill AB (1992) Effects of v-*src* oncogene activated on radiation sensitivity in drug-sensitive and in multidrug-resistant rat fibroblasts. *Radiat Res* **129**: 149–156

Siles E, Villalobos M, Valenzuela MT, Nunez MI, Gordon A, McMillan TJ, Pedraza V and Ruiz de Almodovar JM (1996) Relationship between p53 status and radiosensitivity in human tumour cell lines. *Br J Cancer* **73**: 581–588

Siliciano JD, Canman C, Taya Y, Sakaguchi K, Appella E and Kastan MB (1998) DNA damage induces phosphorylation of the amino-terminus of p53. *Proc 89th AACR* **77**: 527 (abst)

Stewart N, Hicks GG, Paraskevas F and Mowat M (1995) Evidence for a second cell cycle block at G2/M by p53. *Oncogene* **10**: 109–115

Storey A, Thomas M, Kalita A, Harwood C, Gardiol D, Mantovani F, Breuer J, Leigh IM, Matlashewski G and Banks L (1998) Role of a p53 polymorphism in the development of human papilloma-virus-associated cancer. *Nature* **393**: 229–234

Su LN and Little JB (1993) Prolonged cell cycle delay in radioresistant human cell lines transfected with activated ras oncogene and/or simian virus 40 T-antigen. *Radiat Res* **133**: 73–79

Suzuki K, Watanabe M and Miyoshi J (1992) Differences in effects of oncogenes on resistance of gamma rays, ultraviolet light and heat shock. *Radiat Res* **129**: 157–162

Tada M, Matsumoto R, Iggo RD, Onimaru R, Shirato H, Sawamura and Shinohe Y (1998) Selective sensitivity to radiation of cerebral glioblastomas harboring p53 mutations. *Cancer Res* **58**: 1793–1797

Takahashi T, Suzuki H, Hida T, Sekido Y, Ariyoshi Y and Heda R (1991) The p53 gene is very frequently mutated in small-cell lung cancer with a distinct nucleotide substitution pattern. *Oncogene* **6**: 1775–1778

Warenius HM, Browning PG, Britten RA, Peacock JA and Rapp UR (1994) C-*raf*-1 proto-oncogene expression relates to radiosensitivity rather than radioresistance. *Eur J Cancer* **30A**: 369–375

Warenius HM, Jones MD and Thompson CCM (1996) Exit from $G_2$ phase after 2Gy gamma irradiation is faster in radiosensitive human cells with high expression of the RAF-1 proto-oncogene. *Radiat Res* **146**: 485–493

Whitaker SJ, Ung TC and McMillan TJ (1995) DNA double strand break induction and rejoining as determinants of human tumour cell radiosensitivity. A pulsed-field gel electrophoresis study. *Int J Radiat Biol* **67**: 7–18

Xia F, Wang X, Wang YH, Tsang NM, Yandell DW, Kelsey KT and Liber HL (1995) Altered p53 status correlates with differences in sensitivity to radiation-induced mutation and apoptosis in two closely related human lymphoblast lines. *Cancer Res* **55**: 12–15

Zhen W, Denault CM, Loviscek K, Walter S, Geng L and Vaughan AT (1995) The relative radiosensitivity of TK6 and W1-L2-NS lymphoblastoid cells derived from a common source is primarily determined by their p53 mutational status. *Mutat Res* **346**: 85–92

© 2000 Cancer Research Campaign
